# Body image and hopelessness among early‐stage breast cancer survivors after surgery in China: A cross‐sectional study

**DOI:** 10.1002/nop2.884

**Published:** 2021-05-03

**Authors:** Qiong Wu, Yongtian Yin, Qing Wang, Shiyuan Wang, Xinjie Jia

**Affiliations:** ^1^ The Second Surgical Department of Breast Cancer Tianjin Medical University Cancer Institute and Hospital Tianjin China; ^2^ Academic Affairs Office Shandong University of Traditional Chinese Medicine Jinan China; ^3^ Scientific Research Department Shandong University of Traditional Chinese Medicine Jinan China

**Keywords:** body image, breast cancer survivors, China, cross‐sectional study, hopelessness

## Abstract

**Aim:**

To examine the body image and hopelessness status of early‐stage breast cancer survivors who received a one‐time dressing change after surgery and were ready to be discharged from a hospital in China.

**Design:**

A cross‐sectional study.

**Methods:**

Participants were 211 women with breast cancer who had received a one‐time dressing change postsurgery. Spearman's correlation and structural equation modelling were used for data analysis.

**Results:**

Participants in the 35–45 age group and those who underwent bilateral mastectomy presented higher scores for hopelessness. Married participants showed lower scores for the feelings about the future subscale compared to other subscales. Body image was significantly and positively associated with and had a positive effect on hopelessness. Women with a more positive appraisal of their body image tended to report lower levels of hopelessness. Healthcare providers should evaluate patients’ distress levels after a mastectomy to identify women who may require early psychosocial intervention.

## 1 INTRODUCTION

### 1.1 Breast cancer

Worldwide, breast cancer is the most common cancer among women (Ferlay et al., 2015). Based on data from the Global Cancer Statistics 2018, it accounted for nearly 2.1 million newly diagnosed cancer cases in 185 countries (Bray et al., 2018). In China, from 2009 to 2011, breast cancer cases were estimated to be around 15.1% of all newly diagnosed cancer cases (Chen et al., 2016), and it is predicted that 2.5 million new cases of breast cancer will be diagnosed among Chinese women by 2021 (Linos et al., 2008). Particularly, mastectomy has played an important role in this field, and it currently ranks among the most common treatments. In recent reports from Saudi Arabia (Al‐Gaithy et al., 2019) and Brazil (Balabram et al., 2012), its rates ranged from 62.4% to 64.6% of all procedures related to breast cancer in 2011 (Brazil) and 2018 (Saudi Arabia); in some provinces of China (Huang et al., 2016; Lu et al., 2015), these numbers went as high as 80.41%–81.7% in 2014.

Notwithstanding, such surgery procedures often cause many adverse effects towards breast cancer survivors’ physical, psychological and spiritual health in the postsurgery period. Previous studies demonstrated that breast cancer surgery survivors could experience nausea and vomiting (Wesmiller et al., 2017); persistent pain (Ding et al., 2017; Wang et al., 2016); arm dysfunction (i.e. after mastectomy procedures; Crosbie et al., 2010); body image adverse effects (e.g. malformation of one or both breasts and surgery scars; Fouladi et al., 2013); negative emotional responses (e.g. low self‐esteem and moderate or severe anxiety and depression; Ju et al., 2018); and higher levels of anxiety owing to having undergone mastectomy (Kowalczyk et al., 2019).

## 2 BACKGROUND

### 2.1 Hopelessness in cancer patients

Hopelessness can be defined as a cognitive experience in which people feel that their current situation is immutable; hence, they experience negative self‐expectations, pessimistic attitudes about the future and a sense of loss of control, confidence, courage and energy to achieve their own goals in the present and in the future (Alloy et al., 1988; Beck et al., 1996). Moreover, previous studies have shown that hopelessness can cause physical and psychological problems. Cancer patients reported moderate levels of hopelessness, which were shown to correspond to depressive symptoms and maladaptive coping (Grassi et al., 2010). Indeed, a 10‐year longitudinal study among 450 breast cancer survivors pointed out that hopelessness predicts cancer recurrence, reduced survival time (Watson et al., 2005); and another study reported that hopelessness predicts treatment complications (Beck et al., 2010).

Furthermore, several socio‐psychological and physiological factors have been shown to be associated with and affect patients’ hopelessness, such as sleep disorders (Fekih‐Romdhane et al., 2020), lack of perceived social support (Pehlivan et al., 2012) and fear of death and suffering (Shinn et al., 2009). One study among Iranian cancer patients showed that perceived social support was associated with hopelessness levels (Madani et al., 2018). Furthermore, cancer metastasis, pain intensity, anxiety and depression levels were shown to be possible risk factors for higher levels of hopelessness (Yildirim et al., 2009). Hence, hopelessness represents one of the most important psychological consequences of cancer—breast cancer included.

However, most current studies have explored breast cancer survivors’ hopelessness levels after, at least, one month of surgical treatment, and fewer studies have explored their hopelessness levels in between the postsurgery to pre‐discharge periods. Moreover, most current studies chose to explore either socio‐psychological or physical factors of hopelessness (Beck et al., 2010; Grassi et al., 2010; Pehlivan et al., 2012; Watson et al., 2005), and fewer studies have explored the relationship between psychological and physical factors, like body image and hopelessness.

### 2.2 Body image

Body image is a concept that has received many different definitions throughout the years because it is a multidimensional phenomenon that encompasses physiological, psychological and social aspects. Recently, mastectomy has been considered as an amputation, which can be hard for patients owing to its consequences. Particularly, loss of symmetry (caused by the removal of an entire breast) and the obvious change in physical appearance can cause changes in people's body image (Koçan & Gursoy, 2016). Two studies have compared the body image of women who underwent mastectomy with that of those who underwent breast‐conserving therapy, showing that the former experienced worsened body images than the latter (Kwait et al., 2016; Moreira & Canavarro, 2010). In further corroboration, Ardakani et al., (2020) pointed out that, among Iranian women who were undergoing mastectomy, there were those who experienced a disruption in the harmony of their “perfect body” and were shown to have a worsened body image. Besides, a study has shown that women's age may affect their body image distortions after surgery; namely, younger women are more likely to report lower body image perceptions than older women (Paterson et al., 2016).

Previous studies have also identified that a low/negative body image appraisal can lead to many adverse social, physical, psychosocial and mental health consequences. For example, negative body image could trigger physical and emotional changes and may predict diminished life satisfaction (Mincu & Taşcu, 2015). Moreover, breast cancer survivors’ body image disturbances could be associated with a variety of changes they incur after the surgical procedure; for example changes in their interpersonal relationships (Bagheri & Mazaheri, 2015), poor social functioning (Kołodziejczyk & Pawłowski, 2019) and changes in their quality of life, as patients may experience reduced physical health and incur sexual dysfunctions (Bagheri & Mazaheri, 2015; Boquiren et al., 2013; Falk Dahl et al., 2010).

Furthermore, Olasehinde et al., (2019) suggested that body image alterations of young women undergoing mastectomy could interfere with their ability to cope with the psychosocial impacts of the procedure. Additionally, some prior research conducted among breast cancer survivors revealed that body disfiguration (e.g. losing a breast) could cause psychological distress (Falk Dahl et al., 2010; Pierrisnard et al., 2018), undermine their self‐confidence (Rezaei et al., 2016) and become an obstacle to post‐traumatic/postsurgery growth and cognitive functioning (Kołodziejczyk & Pawłowski, 2019). A mixed‐method study reported that women's reactions after seeing their breast site postsurgery were characterized by relief and happiness due to surgery results, powerlessness and distress, thus suggesting a varied and complex set of reactions owing to the procedure (Paraskeva et al., 2019).

Body image has also been related to diminished emotional well‐being in breast cancer survivors. A case‐controlled cohort study compared the body image of breast cancer survivors undergoing mastectomy with that of those undergoing other types of surgery (e.g. breast‐conserving operations or mastectomy with breast reconstruction; Howes et al., 2016); results showed that body image was significantly and positively associated with psychosocial disorder morbidity. Additionally, the mastectomy procedure can result in the total removal of one's breast and leave an ugly scar on the chest causing maladjustment; this was confirmed in a study that showed some survivors might look away from their breasts while changing clothes, not wanting to see their breasts (Kołodziejczyk & Pawłowski, 2019).

### 2.3 Research questions

What is the relationship between body image and hopelessness among breast cancer survivors in the immediate post‐op period after undergoing mastectomy?

### 2.4 Study aims

As aforementioned, hopelessness is a maladaptive psychological response and is a common psychological symptom in cancer patients (Arslan et al., 2009). In this study, we hypothesized that body image changes were threatening events for women, especially for women with breast cancer; they may fail to cope appropriately with the situation owing to the body image alterations they face, which may cause maladaptive psychological responses—namely, hopelessness.

To the best of our knowledge, little research has explored the relationship between body image and hopelessness in breast cancer survivors who underwent a mastectomy, and the relationship between these two constructs during patients’ hospitalization. Instead, most studies focused on long‐term body image, and these have provided us with inconsistent results about body image problems.

Therefore, this study aimed to evaluate the body image and hopelessness status in early‐stage breast cancer survivors who experienced a one‐time dressing change after surgery and were ready to be discharged from a hospital in China.

## 3 METHODS

### 3.1 Design and participants

We used a questionnaire‐based cross‐sectional study design. In the Tianjin Hospital, from October to December 2019, we carried out sample recruitment. The inclusion criteria were (a) aged 18 years or older, (b) women with early‐stage breast cancer (stage I‐III, without metastasis, which was assessed through a histological image analysis), (c) being at the ready‐to‐be‐discharged recovery stage after undergoing a mastectomy. The last inclusion criterion was a very specific one for this study; immediate post‐op period was defined as the period of time during women's surgical recovery in which they had already experienced a one‐time dressing change and had the chance to see the surgical scars over their chest wall. The exclusion criteria were (a) women who underwent breast reconstruction and breast‐conserving therapy; (b) having a severe mental health disorder; (c) not being able to read, understand or speak Chinese; (d) or having any other severe complications.

In total, 230 questionnaires were distributed. Ten questionnaires had more than 50% of missing data, and nine survivors refused to participate. Thence, 211 survivors completed all questionnaires and had their data analysed. The participation rate was 91.7%.

### 3.2 Instruments

#### 3.2.1 Socio‐demographic questionnaire

The socio‐demographic information we collected included patients’ age, occupation, marital status, educational level, illness duration (months) and type of surgery (i.e. unilateral or bilateral mastectomy).

#### 3.2.2 The beck hopelessness scale (BHS)

The Beck Hopelessness Scale (BHS) was designed by Beck et al., (1974). It measures people's hopelessness symptoms and/or negative attitudes about future events. The BHS is a 20‐item self‐reported scale and includes three subscales: feelings about the future (7 items), loss of motivation (11 items) and expectations (2 items). Each item is rated based on binary sentences that are dichotomously coded (true [1] or false [0]); specifically, one point is assigned to items 2, 4, 7, 9, 11, 12, 14, 16, 17, 18, 20 if they are marked true, and one point to items 1, 3, 5, 6, 8, 10, 13, 15, 19 if they are marked false by participants. The total score ranged from 0–20, with higher total scores indicating higher hopelessness levels (scores of 0–3 indicate a minimal, 4–8 a mild, 9–14 a moderate and 15–20 a severe sense of hopelessness). We used its Chinese version, which showed appropriate levels of reliability and validity in a sample of Chinese adolescents (Yuan et al., 2007). In the present study, the Cronbach's alpha for the total scale was 0.843.

#### 3.2.3 The body image after breast cancer questionnaire (BIBCQ)

The BIBCQ was designed by Baxter (1998). It is a multidimensional measure of effect of breast cancer on people's body image. The BIBCQ is a 53‐item scale that has six subscales: vulnerability (12 items), body stigma (16 items), limitations (8 items), body concerns (6 items), transparency (6 items) and arm concerns (5 items). Each item is rated on a 5‐point scale ranging from 1 (strongly disagree/never) to 5 (strongly agree/always). The total score ranged from 53 to 265, with higher total scores indicating more body image problems.

In our study, owing to the inclusion and exclusion criteria (i.e. only patients who underwent a mastectomy were included), we deleted some items about having a lumpectomy, a mastectomy with breast reconstruction and no breast surgical treatment (e.g. items 25–27, 52–53). Thus, the total score of the modified questionnaire ranged from 48 to 240 (48 items). We used its Chinese version, which showed appropriate levels of reliability and validity in a Chinese sample (Zhang et al., 2014). In our study, Cronbach's alpha for the total scale was 0.908.

### 3.3 Statistical Analysis

All statistical analyses were performed using SPSS 22.0 (IBM Corp.). Mean and standard deviations were used to report continuous data, and percentages were used to report categorical data. Kruskal–Wallis and Mann–Whitney tests were used to compare differences in hopelessness by participants’ socio‐demographic characteristics. Spearman's correlation analysis was used to explore the linear correlation between participants’ body image and hopelessness. Structural equation modelling (*SEM*) was used to test the effect of body image on hopelessness while controlling for participants’ socio‐demographic variables. The fit indices for the model were evaluated using the following criteria: *χ* ^2^/*df* < 3, root‐mean‐square error of approximation (RMSEA) < 0.08 and the goodness‐of‐fit index (GFI), adjusted goodness‐of‐fit index (AGFI), comparative fit index (CFI) and Tucker–Lewis index (TLI) > 0.90.

### 3.4 Ethics

First, we sought and obtained ethical approval to conduct this study from the Institutional Review Board of the medical centre. Second, we explained the purpose and procedures of this study to the participants that met the eligibility criteria. Patients who agreed verbally or in writing to participate in this study were, immediately after their consent, asked to complete a self‐reported questionnaire. We reinforced their right to refuse participation at any given time and assured them that we would follow all the required procedures to anonymize their identities.

## 4 RESULTS

### 4.1 Descriptive statistics

Participants’ socio‐demographic characteristics are presented in Table 1. Among 211 participants, the average age was 50.85 years (*SD* = 9.43), ranging from 29 to 77 years; the age group with the highest proportion was the 44–55 years group (35.5%, *n* = 75); most completed primary (34.1%) and secondary school (37.9%); approximately a third (29.9%) were employed and most were married (87.7%).

**TABLE 1:** Descriptive statistics for socio‐demographic characteristics and study variables (*N* = 211)

	*n*	%	*M*	*SD*
Age(year)				
29–35	10	4.7		
35–45	55	26.1		
45–55	75	35.5		
55–77	71	33.6		
Educational level				
Illiterate	17	8.1		
Primary school	72	34.1		
Secondary school	80	37.9		
University/college	42	19.9		
Occupation				
No profession/ /retired	53	25.1		
Worker	42	19.9		
Employee	63	29.9		
Liberal	53	25.1		
Marital status				
Single/divorced/widow	26	12.3		
Married	185	87.7		
illness duration (months)				
<6	178	84.4		
6–12	27	12.8		
<12	6	2.8		
Type of surgery				
Unilateral	189	89.6		
Bilateral	22	10.4		
Body image total			126.95	25.06
Hopelessness total			3.81	3.48
Feelings about the future			0.46	0.89
Loss of motivation			1.73	2.00
Expectations			1.61	1.35
Minimal hopelessness	129	61.1		
Mild hopelessness	51	24.2		
Moderate hopelessness	28	13.3		
Severe hopelessness	3	1.4		

Moreover, participants who had been diagnosed with breast cancer for less than 6 months accounted for 84.4% of the total sample, and 89.6% had a unilateral mastectomy. Their mean body image score was 126.95 (*SD* = 25.06), and the mean hopelessness score was 3.81 (*SD* = 3.48). Most participants reported minimal (0–3 scores) hopeless scores, accounting for 61.1% of the total sample.

Participants’ scores in the BHS scale according to the three subscales and their socio‐demographic characteristics are presented in Table 2. By comparing the means using Kruskal‐Wallis and Mann‐Whitney tests, participants who were in the 35–45 age group presented higher scores for hopelessness (*χ* ^2^ = 8.272, *p* = .041), higher scores for the loss of motivation (*χ* ^2^ = 9.568, *p* = .023) and higher scores for the expectations subscale (*χ* ^2^ = 8.174, *p* = .043); participants who underwent bilateral mastectomy presented higher scores for hopelessness (*Z* = −2.519, *p* = .012), higher scores for the feelings about the future (*Z* = −3.129, *p* = .002) and higher scores for the expectations subscale (*Z* = −2.229, *p* = .026); married participants showed lower scores for the feelings about the future subscale compared to other subscales (*Z* = −2.327, *p* = .020). There were no other significant differences in participants’ socio‐demographic factors.

**TABLE 2:** The distribution of participants’ scores in the Beck Hopelessness Scale by socio‐demographic factors (*N* = 211)

	Hopelessness	Feelings about the future	Loss of motivation	Expectations
Age(year)				
29–35	93.15	101.30	96.90	100.15
35–45	118.24	103.49	116.45	121.29
45–55	91.39	107.79	89.95	92.09
55–77	113.77	106.71	116.14	109.67
*χ* ^2^	8.272	0.350	9.568	8.174
*df*	3	3	3	3
*P*	0.041	0.950	0.023	0.043
Educational level				
Illiterate	113.53	104.03	121.29	102.68
Primary school	110.40	104.31	108.05	114.94
Secondary school	106.81	107.44	104.68	106.70
University/college	93.88	106.95	98.81	90.68
*χ* ^2^	2.350	0.198	1.884	4.519
*df*	3	3	3	3
*P*	0.503	0.978	0.597	0.211
Occupation				
No profession/retired	114.26	110.93	112.60	111.49
Worker	118.67	112.12	111.94	119.98
Employee	100.97	103.68	105.63	97.45
Liberal	93.68	98.97	95.13	99.59
*χ* ^2^	5.481	2.413	2.877	4.728
*df*	3	3	3	3
*P*	0.140	0.491	0.411	0.193
Marital status				
Single/ Divorced/Widow	119.13	126.98	120.40	111.44
Married	104.15	103.05	103.98	105.24
Z	−1.184	−2.327	−1.326	−0.500
*P*	0.236	0.020	0.185	0.617
Illness duration (months)				
<6	103.92	103.27	105.05	102.58
≥6	96.94	101.20	89.50	105.76
Z	−0.575	−0.208	−1.310	−0.267
*P*	0.565	0.835	0.190	0.789
Type of surgery				
Unilateral	102.43	102.39	103.79	102.90
Bilateral	136.70	137.00	124.98	132.64
Z	−2.519	−3.129	−1.590	−2.229
*P*	0.012	0.002	0.112	0.026

### 4.2 Correlations between body image and hopelessness

Table 3 presents the results of Spearman's correlation analysis. The results showed that body image was significantly and positively associated with hopelessness (*r* = 0.423, *p* < .01) and its subscales (*r* = 0.202 ~ 0.413, all *p* < .01).

**TABLE 3:** Correlations for all variables (*N* = 211)

	Hopelessness total	Feelings about the future	Loss of motivation	Expectations
Body Image total	0.423**	0.202**	0.326**	0.413**
Vulnerability	0.422**	0.233**	0.297**	0.431**
Body Stigma	0.386**	0.160*	0.290**	0.370**
Limitations	0.374**	0.266**	0.323**	0.341**
Body Concerns	0.063	0.102	0.066	0.019
Transparency	0.386**	0.088	0.300**	0.378**
Arm Concerns	0.159*	0.061	0.120	0.161*

* *p <* .05;

** *p <* .01.

### 4.3 Regression analyses of body image on hopelessness

**FIGURE 1:**
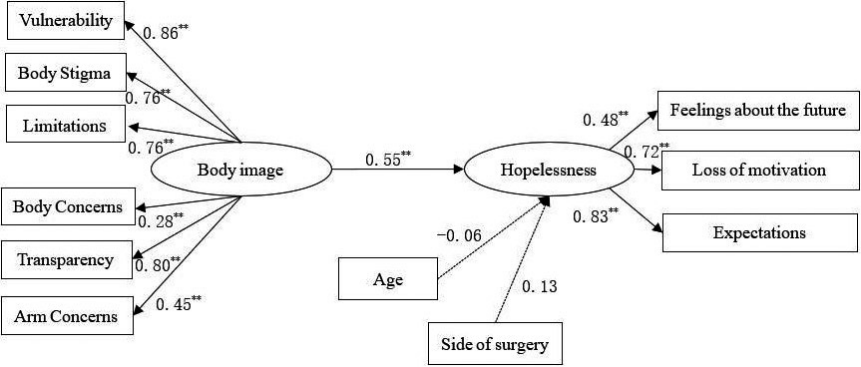
Structural equation model of the effect of body image on hopelessness (*N* = 211). Standardized estimates for significant paths at *p* < .001 are presented. Non‐significant paths are shown with dotted lines

Figure 1 presents the results of the *SEM* to test the effect of body image on hopelessness. Owing to consideration of the covariance parameters problem, these were included in the model. The fit indices for the model were *χ* ^2^ = 61.385, *df* = 38, *χ* ^2^ */df* = 1.615 < 3, RMSEA = 0.054 < 0.08, GFI = 0.952, AGFI = 0.916, CFI = 0.968 and TLI = 0.954. The results suggested that body image had a positive effect on hopelessness (*β* = 0.152, *p* < .001).

## 5 DISCUSSION

### 5.1 Assessment of body image and hopelessness

Our results showed that participants had a mean hopelessness score of 3.81 ± 3.48; it was lower than that of Iranian breast cancer patients in a previous study (4.06 ± 2.67) (Madani et al., 2018) and lower than that of Adana/Turkey breast cancer patients (5.49 ± 3.80) (Öztunç et al., 2013). The discrepancies in the results of these studies compared to ours can be explained by sample differences. In the present study, participants were at an early stage of breast cancer, whereas participants in Madani et al. and’s (2018) study were at the end stage of breast cancer and those in Öztunç et al. and’s (2013) study were undergoing chemotherapy. A previous study has shown that patients in palliative care might experience fear of a painful death, poor symptom management or abandonment, which are all related to hopelessness (Breitbart et al., 1998). Hence, it corroborates our assertion about sample differences; the cited studies had patients that were already under palliative care, and they indeed showed higher hopelessness scores.

Additionally, our results showed that 61.1% of our sample had a minimal sense of hopelessness; this indicates that, during the immediate post‐op period (after a one‐time dressing change and close to discharge from hospital stay), our sample experienced a relatively low sense of hopelessness. Thus, future longitudinal studies are warranted to explore the variance in breast cancer survivors’ hopelessness levels after surgery.

Our results also showed that participants’ mean body image score was 126.95 ± 25.06; these scores were lower than those of a previous study conducted among breast cancer survivors on body image (137 ± 16.75) (Kowalczyk et al., 2019). This difference could owe to the inclusion criteria of the cited study which included those undergoing breast‐conserving therapy (Kowalczyk et al., 2019). Another explanation could be that, in our study, the survivors, who had just completed the operation, were more likely to pay attention to postoperative rehabilitation or to the cancer that had finally been removed, rather than care about bodily changes. A longitudinal study found that women's body image was worse 6 to 7 months after surgery (Moreira & Canavarro, 2010). Thus, although women's body image immediately after surgery may, indeed, not be affected that much, it may change in the long‐term. This might owe to the high levels of stress they experience as a consequence of the cancer diagnosis and the need to undergo surgery. Thus, future studies are warranted to further analyse these specific topics (e.g. worsened body image after mastectomy in the long‐term owing to high levels of stress caused by cancer diagnosis).

In our study, results demonstrated that body image was significantly and positively associated with and had a positive effect on hopelessness. This indicates that, although breast cancer survivors in the immediate post‐op period can withstand the changes that occur to their bodies—owing to surgery—and their accompanying negative emotions, women that pay more attention to their body image may be more likely to experience higher hopelessness levels; thus, efforts should be made to identify such patients.

### 5.2 Relationship between hopelessness and the study variables

Our results showed that survivors of bilateral mastectomy had higher hopelessness scores owing to the feelings about the future and expectations subscales. We speculated that, compared to survivors who underwent a unilateral mastectomy, those who underwent a bilateral mastectomy might have thought that their health condition was more serious; moreover, they could have been informed by healthcare providers about experiencing severe postoperative symptoms as a result of this procedure (e.g. increased risk for bilateral lymphoedema). Such negative outcomes, in turn, might disrupt Chinese women's common daily routine in the near future (e.g. daily work) causing them to experience more negative feelings about their future and lower expectations compared to unilateral breast cancer survivors, justifying these high scores.

Additionally, single/divorced/widowed participants showed higher scores for the feelings about the future subscale (i.e. lower expectancies about the future) compared to married participants. This may be because of differences in access to social support for each of these socio‐demographic groups; women in such circumstances need to cope with the emotional effects of the illness and of the procedures undertaken, and being married denotes that they have better access to social support during this difficult time (e.g. from their husbands). Conversely, single/divorced/widowed women may have more difficulties in accessing such support. However, most studies do not find statistical differences about this subscale when comparing it by marital status (Avci et al., 2009; Yildirim et al., 2009). Still, one study by Sahin et al., (2013), which analysed the hopelessness levels of women who had terminal cancer, showed that married patients’ hopelessness scores were higher than those of single patients. This between‐study difference may be explained by the two samples being at different stages of a similar disease. In Şahin et al., (2013)’s study, participants had terminal cancer, which could have led them to worry more about their partners’ lives after they passed away. In contrast, our participants had recently undergone a mastectomy, so they might have needed company to share their fears about the situation. Such support may have been associated with their less impaired thoughts about their future.

Our results also showed that higher scores for hopelessness, loss of motivation and expectations were significantly associated with the 35–45 age group. Contrariwise, Gumus et al., (2011) showed, in a sample of women with breast cancer, that there was no significant correlation between participants’ age and their hopelessness score (*p* = .404); this was corroborated by Öztunç et al., (2013) who pointed out that there was no statistically significant difference between breast cancer patients’ age and their hopelessness scores. This might be because most Chinese patients who were aged 35–45 in our sample were both at the peak of their careers and at a stage where they needed to take care of their families. After surgery, they might have found themselves faced with various uncertainties and hindrances, such as those regarding their health (e.g. the development of another breast cancer and the cure rate), their work (e.g. whether they will be able to adapt to work) and their families (e.g. the couple's relationship or marital satisfaction). Such concerns may have resulted in the observed higher hopelessness, loss of motivation and lower expectations towards the future.

Finally, we found no significant differences between other socio‐demographic characteristics (e.g. educational level, occupation and illness duration) and hopelessness. This is corroborated by Avci et al., (2009) who found that education and employment did not affect hopelessness levels among breast cancer survivors after mastectomy. However, Gumus et al., (2011) showed a negative correlation between illness duration and hopelessness scores in women with breast cancer who began chemotherapy and/or radiotherapy after about a month of the total healing of their surgical wounds. The difference between our results and those of Gumus et al., (2011) may be related to our sample having received only the early surgical treatment for cancer (not any type of radio‐ or chemotherapy); this means that the treatment for participants in Gumus et al.’s study was less likely to take up a lot of their time and energy and to be associated with higher hopelessness scores. Moreover, another explanation may be the wider range of side effects that occur owing to chemotherapy and/or radiotherapy, which may impact other factors that, in turn, are associated with hopelessness. Therefore, future longitudinal and randomized controlled trial studies are warranted to analyse the long‐term relationship between these variables and their causality.

## 6 LIMITATIONS

First, we did not include more detailed socio‐demographic variables to be compared with breast cancer survivors’ body image and hopelessness (e.g. caregivers’ attitude and behaviour); hence, future studies are warranted to compare these interest variables with a greater number of socio‐demographic characteristics.

Second, the current study only investigated participants’ body image and hopelessness during a very specific period of time, and it used a cross‐sectional design; thus, generalizations should be made with caution, and our results do not denote any type of causality. Future longitudinal studies are warranted to clarify the fluctuations in body image and hopelessness scores of the studied sample over time.

Third, our sample size was relatively small and a power calculation was not initially conducted; thus, future research with larger samples and power calculations before sample recruitment are warranted.

Fourth, although the current study excluded patients with clinically diagnosed mental health problems, we did not use any type of measurement for depressive symptoms and anxiety levels, and these were not used as possible covariates; thus, future studies are warranted to analyse these covariates and assess for these psychological problems upon sample recruitment.

## 7 CLINICAL IMPLICATIONS

Our most meaningful result was that, although we did not have baseline information on our sample's body images prior to the surgery, breast cancer survivors’ bodily changes related to the surgical procedure may be associated with an increase in their body image scores—denoting higher body image problems. Moreover, such an increase was associated with increased hopelessness. These findings indicated that healthcare providers should pay attention when evaluating patients’ distress levels after a mastectomy procedure, as this may allow for identifying the most important issues they face after surgery. For example, rehabilitation exercises could improve breast cancer survivors’ upper limb function (Hong & Hui‐ling, 2010), which may increase their positive feelings about the future and motivation. Additionally, healthcare practitioners should endeavour to provide patients with more information amid their treatments, so that patients can have more tools to deal with the negative outcomes related to cancer treatment procedures. A more positive approach to dealing with the issues that come with cancer may help women who undergo mastectomy to have better body image and lower hopelessness in the short‐term postoperative recovery period.

## 8 CONCLUSION

Body image was significantly and positively associated with and had a positive effect on hopelessness among early‐stage breast cancer survivors in China. These findings suggest that breast cancer survivors’ positive reappraisal of body image may be associated with reduced hopelessness levels. However, the underlying mechanism of these associations is still poorly understood and warrants further study.

## CONFLICT OF INTEREST

The authors have declared no conflicts of interest.

## AUTHOR CONTRIBUTIONS

Qiong W. and Y.Y. contributed to conceptualization; Qiong W. and Y.Y. contributed to methodology; Qiong W. contributed to software; Qiong W. and Y.Y. contributed to validation; Qiong W. contributed to formal analysis; Qing W. and X.J. contributed to investigation; Qing W. and X.J. contributed to resources; Qiong W. contributed to data curation; Qiong W., Y.Y. and S.W. contributed to writing—original draft preparation; Y.Y. and Qing W. contributed to writing—review and editing; Qiong W. and S.W. contributed to visualization; Y.Y. and S.W. contributed to supervision; Y.Y. contributed to project administration; and Y.Y. contributed to funding acquisition.

## Data Availability

Data available on request from the authors. The data that support the findings of this study are available from the corresponding author upon reasonable request.
